# Investigating and analyzing the current situation and factors influencing chronic neck, shoulder, and lumbar back pain among medical personnel after the epidemic

**DOI:** 10.1186/s12891-024-07425-x

**Published:** 2024-04-23

**Authors:** Ansu Wang, Yufeng Zhou, Xuyan Li, Weiqun Wang, Xu Zhao, Ping Chen, Wenbo Liao

**Affiliations:** Department of Orthopedics, Affiliated Hospital of Zunyi Medical University, Zunyi, 563000 Guizhou Province China

**Keywords:** Medical staff, Chronic neck and shoulder pain, Chronic lumbar back pain, Influencing factors

## Abstract

**Background:**

Chronic shoulder and neck pain is one of the most common chronic occupational disorders, with an average incidence rate of 48.5%, severely affecting patients’ quality of life and ability to work. According to epidemiological research, the prevalence of chronic neck, shoulder, and low back pain in adults over the age of 45 ranges from 40 to 80%. According to reports, medical staff have a higher incidence rate than other populations, and there is a positive correlation between the grade of the medical institution and the incidence rate, making medical staff a priority group for the prevention of chronic neck, shoulder, and low back pain. By the end of 2022, China has been fully opened to epidemic prevention and control, the total number of patients in domestic hospitals has increased significantly, and resulting in medical personnel shoulting great pressure, which seriously affects the physical and mental health of medical personnel. The aim of this study was to explore the risk factors of chronic neck, shoulder and lumbar back pain in medical staff. To provide guidelines for medical staff to improve cervical and lumbar subacute pain and reduce the emergence of spinal lesions.

**Methods:**

From January to February 2023, 602 staff members of a third-grade hospital in Zunyi City were studied by Questionnaire star. Univariate and multivariate Logistic regression were used to analyze the independent risk factors of chronic neck, shoulder and lumbar back pain in medical staff, with stepwise regression utilized to choose the optimum model. The model was selected using Akaike’s information criterion (AIC) and the Hosmer-Lemeshow goodness-of-fit test.

**Results:**

A total of 602 medical staff were polled, and the findings revealed that 588 cases of chronic neck, shoulder, and low back pain of varied severity had occurred in the previous 1 to 2 years, with a 97.7% incidence rate; logistic regression analysis revealed that anxiety level, frequency of bending over in the previous 1 to 2 years, whether related preventive measures were taken at work, gender, positive senior title, daily ambulation time, and whether the department they worked in organized independent influencing factors.

**Conclusion:**

The incidence of chronic neck, shoulder, and lumbar back pain among medical staff is high; its influencing factors are different and have not been systematically identified. Hospitals should take effective measures tailored to local conditions to improve the physical and mental health of medical staff.

## Background

Chronic neck, shoulder, and lumbar back pain refer to a clinical condition in which chronic pain and impairment of mobility occur in the neck, shoulder, back, lumbar, or upper buttocks. It can manifest as cervical spondylosis, scapulohumeral periarthritis, lumbar spondylosis, lumbar muscle strain, and back myofascitis [1, 2]. Chronic neck and shoulder pain is a common occupational disease in clinical practice and adversely affects the quality of life and daily work of patients. It has an average morbidity of 48.5% [3, 4]. In recent years, due to the rapid development of the medical and healthcare industry in China, the competition in this industry has increased. After the outbreak of the COVID-19 pandemic at the end of 2019, medical personnel are not only burdened with clinical, teaching, and research-related activities, but they are also responsible for preventing and controlling the epidemic. At the end of 2022, after the opening of national epidemic prevention and control measures, the number of patients in hospitals increased substantially, but there was a shortage of medical personnel, which greatly increased the number of working hours and their social, professional, and personal pressure and adversely affected their physical and psychological health. An epidemiological study showed, that the incidence of cervical spondylosis and lumbar spondylosis has increased in recent years, especially among people who are over 45 years old. The incidence rate of chronic neck, shoulder, and lumbar back pain is as high as 40–80%, and10–15% of the patients become disabled due to this condition [5, 6]. Some studies [1, 7], have found that the rate of incidence of neck, shoulder, and lumbar back pain among people working in medical institutions is higher than that of people working in other places, and a positive correlation was found between the level of the medical institutions and the rate of incidence. Thus, neck, shoulder, and lumbar back pain of medical personnel need to be prevented. Chronic neck, and shoulder pain can severely affect the daily life and the quality of life of patients, the key to relieving pain and treating the disease is to take timely standard prevention measures. In this study, we assessed the current situation and factors influencing chronic neck, shoulder, and lumbar back pain among medical personnel. The purpose of this study is providing a reference for medical personnel to effectively improve the condition of their neck and lumbar and also decrease the incidence of cervical spondylosis and lumbar spondylosis.

## Participants and methods

### Participants recruited in the study

From January to February 2023, 602 during service staff members from a class A tertiary hospital in Zunyi City were selected as participants based on a simple random sampling method. The inclusion criteria were as follows: (1) Doctors, nurses, and personnel from other departments with work experience of at least one year and aged ≥ 18 years. (2) Those who were on the job when the study was conducted. (3) A maximum of 40 h of work per week. 4.Individuals who provided informed consent and volunteered to participate in the study. The exclusion criteria were as follows: (1) Individuals who were engaged in advanced studies and trainees. (2) Individuals with cognitive impairment. 3.Those who experienced significant changes in their family or took antipsychotic or neurological medication recently. 4. Individuals who had experienced cervical, thoracic, or lumbar vertebral fractures or other spinal surgeries, as well as, other major surgeries. 5. Individuals who were diagnosed with pelvic disease, prostate disease, liver, kidney, spleen and heart disease, or pregnant. This study was approved by the Ethics Committee of Zunyi Medical University before it was conducted.

### Research tools

Based on the methods described in other studies [8, 9] and job category in hospitals, we used “A questionnaire of chronic neck, shoulder, and lumbar back pain among medical personnel” to collect the data; the Cronbach’s α index was 0.86, and the internal consistency was high. The main content of the questionnaire was as follows: (1) The demographic information of medical personnel included details on their gender, age, height, weight, profession, education background, length of service, marriage, professional title, presence/absence of underlying medical conditions, etc. (2) Information on potential risks, including having children or not; frequency of having neck, shoulder, and lumbar back pain in the past year; being/not being responsible for household chores; extent of anxiety; work-related pressure on medical personnel; job satisfaction; whether taking relevant measures to prevent neck, shoulder, and lumbar back pain at work; knowledge about preventing neck, shoulder, and lumbar back pain; whether they underwent training to prevent neck, shoulder, and lumbar back pain in their departments; whether they exercised frequently; frequency of bending over in the past 1–2 years; the time spent working at a desk each day with the head tilted down; the time spent sitting every day; the number of times they moved or lifted instruments and equipment every day; the intensity of work each day; the time spent looking at phones each day, etc. (3) The Oswestry disability index (ODI) [10]; the ODI consisted of 10 items, including pain intensity, daily activities, lifting, walking, sitting, standing, sleeping, sexual activity, social activity, and traveling.

### Data collection

The in-service medical workers were surveyed using a questionnaire. They were requested to submit the questionnaire only after answering all questions; each participant could fill out the form only once. The participants were given 10 min to answer all questions in the questionnaire. All participants were anonymous, and we received 602 valid questionnaires. The participants were divided into the case group and the control group based on whether they experienced neck, shoulder, and lumbar back pain frequently in the last 1–2 years. Criteria for inclusion in the case group: In the last 1–2 years, there were at least four incidences of stiffness, fatigue, soreness, numbness, and other feelings of discomfort associated with the neck, shoulders, back, lumbar, and upper buttocks, which lasted for more than two weeks and occurred repeatedly [11].

### Statistical analysis

Single-factor analysis was performed using the R statistical software (version: 4.2.2). Measurement data are presented as the mean ± standard deviation ($\stackrel{-}{x}\pm s$) or median (Q1, Q3), whereas, enumeration data are presented as the sample size and percentage. Measurement data were analyzed by the Wilcoxon rank sum test or the Kruskal-Wallis test. Enumeration data were analyzed by the Chi-squared test or Fisher’s exact test. We also performed. Pearson’s correlation coefficient and Spearman’s correlation coefficient. Single-factor and multi-factor logistic regression analyses were performed. Relevant factors were identified in the single-factor logistic regression model, and they were used to construct the multi-factor logistic regression model. The best model was obtained by performing stepwise regression. Akaike’s information criterion (AIC) was used as the selection criterion for the model; the lower the AIC value, the better the corresponding model. The Hosmer-Leme show test was performed to evaluate the goodness of fit; a significant difference, indicated a poor model fit. All results were considered to be significant at α = 0.05.

## Results

### Comparison of the general data of the participants

We found significant differences (*P* < 0.05) between the groups concerning gender; profession; frequency of bending over in the past 1–2 years; the time spent working at a desk each day with the head tilted down; the number of times they moved or lifted instruments and equipment every day; working intensity (total rest time every day, including sleeping time; whether they took relevant measures to prevent neck, shoulder, and lumbar back pain; whether they underwent training to prevent neck, shoulder, and lumbar back pain in their departments; anxiety about; work-related pressure. Other factors were not significantly different; the details are presented in Table 1.

**Table 1:** Comparison of the basic characteristics between the case and control groups [sample size, M (P25, P75)]

Variate	Classification	Control group	Case group	Statistical magnitude	P value
Age		319,37.00(32.00,42.00)	265 37.00(33.00,41.00)	42692.5	0.834^
Height		323160.00(157.00,165.00)	265160.00(158.00,163.00)	43891.5	0.592^
Weight		322 56.00(50.00,65.00)	266 57.00(51.00,63.00)	43542.5	0.726^
BMI value		321 22.31(20.31,24.14)	265 21.87(20.28,24.03)	43814.5	0.53^
Gender	Female	242(73.33%)	236(86.76%)		< 0.001
	Male	88(26.67%)	36(13.24%)		
Profession	Nurse	205(62.12%)	202(74.26%)	10.04	0.007*
	Doctor	91(27.58%)	51(18.75%)		
	Other workers	34(10.30%)	19(6.99%)		
Marriage	Married	276(83.64%)	235(86.40%)	3.613	0.164*
	Single	51(15.45%)	31(11.40%)		
	Divorced	3(0.91%)	6(2.21%)		
Having children	No	62(18.79%)	44(16.18%)	0.93	0.628*
	1	140(42.42%)	124(45.59%)		
	2 and more	128(38.79%)	104(38.24%)		
Being responsible for household chores	No	175(53.03%)	144(52.94%)	0	1
	Yes	155(46.97%)	128(47.06%)		
Educational background	College and technical school graduates	8(2.42%)	11(4.04%)	5.888	0.117*
	Bachelor	225(68.18%)	203(74.63%)		
	Master	79(23.94%)	47(17.28%)		
	Doctor	18(5.45%)	11(4.04%)		
Length of service	1year∼	200(60.61%)	168(61.76%)		0.801
	15 year∼	130(39.39%)	104(38.24%)		
Professional title	Junior	106(32.12%)	102(37.50%)	9.301	0.054*
	Intermediate	128(38.79%)	104(38.24%)		
	Deputy senior	71(21.52%)	52(19.12%)		
	Senior	13(3.94%)	13(4.78%)		
	Others	12(3.64%)	1(0.37%)		
Frequency of bending over in the past 1–2 years	Low	178(53.94%)	42(15.44%)		< 0.001
	High	152(46.06%)	230(84.56%)		
The amount of time spent working with the head down at a desk each day	<6 h/day	206(62.42%)	133(48.90%)		< 0.001
	≥ 6 h/day	124(37.58%)	139(51.10%)		
The amount of time sitting every day	<6 h/day	223(67.58%)	164(60.29%)		0.073
	≥ 6 h/day	107(32.42%)	108(39.71%)		
Times of moving or lifting instruments and equipment every day	<6 times/day	264(80.00%)	188(69.12%)		0.002
	≥ 6 times/day	66(20.00%)	84(30.88%)		
Working intensity each day (total rest time every day, while sleeping time not included)	≥ 4 h/day	218(66.06%)	156(57.35%)		0.035
	<4 h/day	112(33.94%)	116(42.65%)		
Taking relevant measures to prevent neck, shoulder, and lumbar back pain	No	258(78.18%)	187(68.75%)		0.009
	Yes	72(21.82%)	85(31.25%)		
Knowing how to prevent neck, shoulder, and lumbar back pain	No	262(79.39%)	215(79.04%)		0.92
	Yes	68(20.61%)	57(20.96%)		
Conducting training to prevent neck, shoulder, and lumbar back pain in their department	No	273(82.73%)	250(91.91%)		< 0.001
	Yes	57(17.27%)	22(8.09%)		
Extent of anxiety	Low	314(95.15%)	155(56.99%)		< 0.001
	High	16(4.85%)	117(43.01%)		
Extent of working pressure	Low	178(53.94%)	121(44.49%)		0.022
	High	152(46.06%)	151(55.51%)		
Job satisfaction	Low	182(55.15%)	170(62.50%)		0.081
	High	148(44.85%)	102(37.50%)		
Exercise frequency	Low	233(70.61%)	211(77.57%)		0.063
	High	97(29.39%)	61(22.43%)		
The amount of time spent looking down at phones each day	<6 h/day	316(95.76%)	254(93.38%)		0.206
	≥ 6 h/day	14(4.24%)	18(6.62%)		
With underlying medical conditions (High blood pressure, diabetes, coronary heart disease? )	No	300(90.91%)	249(91.54%)	0.772	0.680*
	1 condition	25(7.58%)	17(6.25%)		
	2 conditions	5(1.52%)	6(2.21%)		

*^P* value was calculated by the Wilcoxon rank sum test*P value was calculated by the Chi-squared test, and others are calculated by Fisher’s exact test

**Correlation analysis between the frequency of chronic neck, shoulder, and lumbar back pain and ODI scores**.

**Fig. 1:**
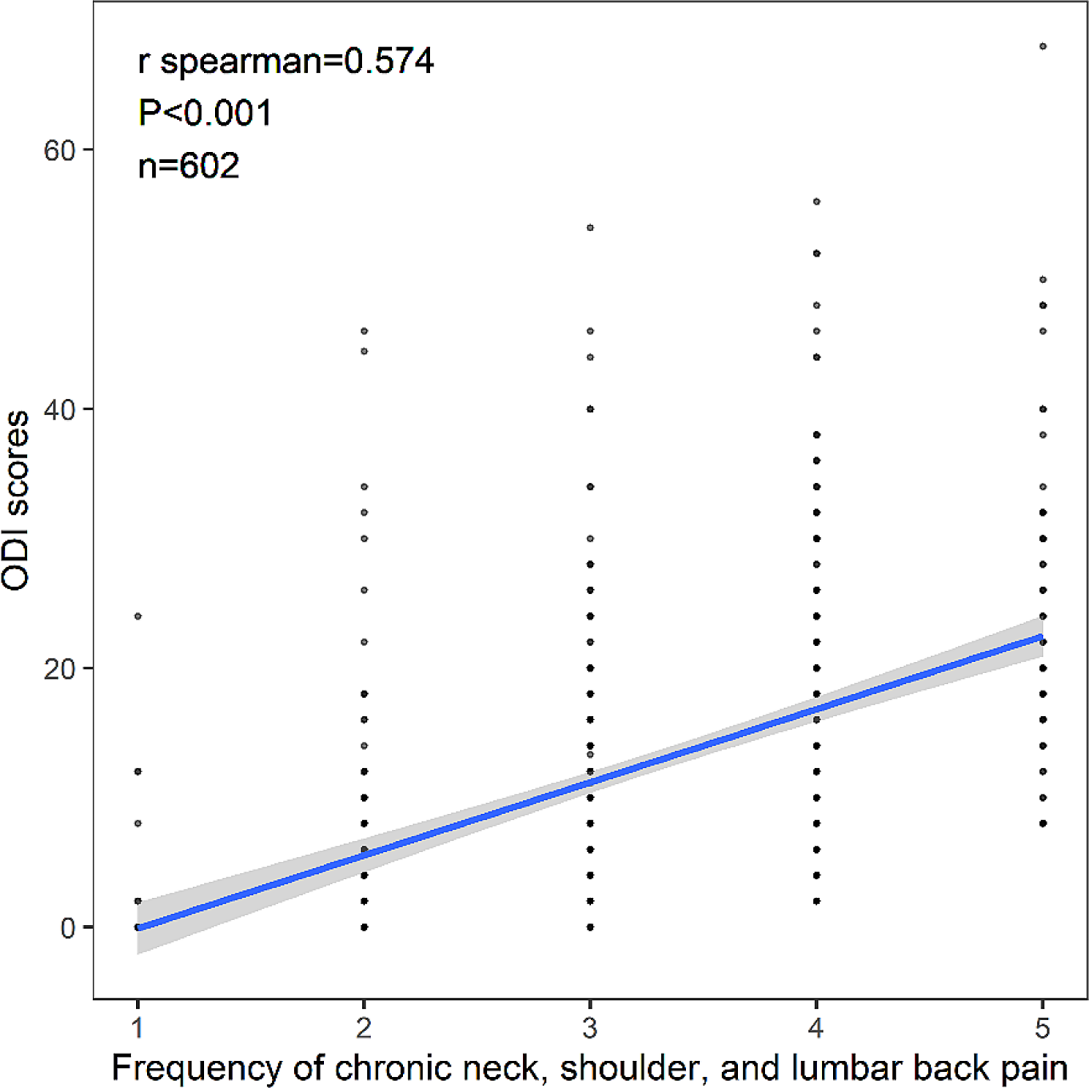
Correlation between ODI scores and the frequency of chronic neck, shoulder, and lumbar back pain

The correlation coefficient for the correlation between the ODI score and the frequency of chronic neck, shoulder, and lumbar back pain (rated on a scale of 1–5) was *r* = 0.574 (*P* < 0.001). The details are presented in Table 2; Fig. 1.

**Table 2:** Correlation between chronic neck, shoulder, and lumbar back pain and ODI scores

Variate	Sample size	Mean ± standard	Median(Q1,Q3)	min	max
Extent of pain	602	1.53 ±0.90	1.00(1.00, 2.00)	0	5
Activities of daily living	602	0.50 ±0.63	0.00(0.00, 1.00)	0	4
Lifting	602	0.78 ±0.94	0.00(0.00, 1.00)	0	4
Walking	602	0.26 ±0.84	0.00(0.00, 0.00)	0	5
Sitting	602	0.92 ±1.03	1.00(0.00, 1.00)	0	4
Standing	602	0.88 ±0.87	1.00(0.00, 1.00)	0	5
Sleeping	602	0.29 ±0.78	0.00(0.00, 0.00)	0	5
Sexual activity	602	0.37 ±0.92	0.00(0.00, 0.00)	0	5
Social activity	598	0.42 ±0.72	0.00(0.00, 1.00)	0	5
Traveling	602	0.50 ±0.76	0.00(0.00, 1.00)	0	5
Total	602	12.92 ±11.21	10.00(4.00, 18.00)	0	68
Frequency of chronic neck, shoulder, and lumbar back pain	602	3.31 ±0.97	3.00(3.00, 4.00)	1	5

### Single-factor analysis of chronic neck, shoulder, lumbar, and back pain in medical personnel

We conducted a single-factor logistic regression analysis with the occurrence of chronic neck, shoulder, lumbar, and back pain in the past 1–2 years as the dependent variable. The findings indicated that the following factors were independent risk factors for chronic neck, shoulder, and low back pain among medical personnel: gender, job title, frequency of bending over in the past 1–2 years, time spent working with head down, number of times moving or lifting instruments per day, work intensity (cumulative rest time per day, excluding sleep time), whether or not measures were taken to prevent neck, shoulder, and low back pain, whether or not training on neck, shoulder, and low back pain protection was organized by the department (or unit) where they were working, anxiety about pain, and work stress. Detailed information can be found in Table 3.

**Table 3:** The results of the single-factorlogistic regression analysis

Variate		Regression coefficient	Standard error	Z value	P value	OR (95%CI)
Gender	Female	0.869	0.218	3.985	< 0.001	2.384 (1.555–3.654)
Profession	Doctor	-0.564	0.201	-2.807	0.005	0.569 (0.384–0.843)
	Other workers	-0.567	0.303	-1.871	0.061	0.567 (0.313–1.027)
Marriage	Married	0.337	0.244	1.379	0.168	1.401 (0.868–2.262)
	Divorced	1.191	0.743	1.603	0.109	3.290 (0.767–14.112)
Having children	1	0.222	0.233	0.953	0.341	1.248 (0.791–1.969)
	2 and more	0.135	0.237	0.570	0.568	1.145 (0.719–1.823)
Be responsible for household chores	yes	0.004	0.164	0.022	0.983	1.004 (0.728–1.384)
Educational background	Bachelor	-0.421	0.475	-0.888	0.375	0.656 (0.259–1.663)
	Master	-0.838	0.500	-1.676	0.094	0.433 (0.162–1.152)
	Doctor	-0.811	0.602	-1.347	0.178	0.444 (0.137–1.446)
Length of service	15 years∼	-0.049	0.168	-0.290	0.772	0.952 (0.685–1.324)
Professional title	Intermediate	-0.169	0.191	-0.883	0.377	0.844 (0.580–1.229)
	Deputy senior	-0.273	0.229	-1.191	0.234	0.761 (0.486–1.193)
	Senior	0.038	0.416	0.092	0.926	1.039 (0.460–2.349)
	Others	-2.446	1.049	-2.332	0.020	0.087 (0.011–0.677)
Frequency of having neck, shoulder, lumbar, and back pain in the past 1–2 years	High	53.132	29164.740	0.002	0.999	118848598931271590088068.000(0.000-Inf)
Frequency of bending over in the past 1–2 years	High	1.858	0.201	9.251	< 0.001	6.413 (4.326–9.507)
The amount of time spent working with the head down at a desk each day	≥ 6 h/day	0.552	0.166	3.319	< 0.001	1.736 (1.253–2.405)
The amount of time sitting every day	≥ 6 h/day	0.317	0.171	1.853	0.064	1.372 (0.982–1.918)
Times of moving or lifting instruments and equipment every day	≥ 6 times/day	0.581	0.190	3.053	0.002	1.787 (1.231–2.594)
Working intensity each day (total rest time every day, while sleeping time not included)	<4 h/day	0.370	0.169	2.188	0.029	1.447 (1.039–2.016)
Taking relevant measures to prevent neck, shoulder, and lumbar back pain	yes	0.488	0.187	2.612	0.009	1.629 (1.130–2.349)
Knowing how to prevent neck, shoulder, and lumbar back pain	yes	0.021	0.202	0.105	0.916	1.021 (0.688–1.517)
Conducting training to prevent neck, shoulder, and lumbar back pain in their department	yes	-0.864	0.266	-3.250	0.001	0.421 (0.250–0.710)
Extent of anxiety	High	2.696	0.284	9.490	< 0.001	14.814 (8.490–25.849)
Extent of working pressure	High	0.379	0.165	2.305	0.021	1.461 (1.058–2.018)
Job satisfaction	High	-0.304	0.167	-1.819	0.069	0.738 (0.532–1.024)
Exercise frequency	High	-0.365	0.189	-1.929	0.054	0.694 (0.479–1.006)
The amount of time spent looking down at phones each day	≥ 6 h/day	0.470	0.366	1.283	0.200	1.600 (0.780–3.279)
With underlying medical conditions (High blood pressure, diabetes, coronary heart disease? )	1 condition	-0.199	0.326	-0.612	0.541	0.819 (0.433–1.552)
	2 conditions	0.369	0.612	0.603	0.547	1.446 (0.436–4.794)
Age		-0.004	0.010	-0.468	0.640	0.996 (0.977–1.014)
Height		-0.019	0.014	-1.382	0.167	0.981 (0.955–1.008)
Weight		-0.006	0.007	-0.930	0.353	0.994 (0.981–1.007)
BMI value		-0.011	0.015	-0.743	0.457	0.989 (0.960–1.018)

### Multiple-factor analysis of chronic neck, shoulder, lumbar, and back pain among participants

We performed a multiple-factor binary logistic regression analysis with the frequent occurrence of chronic neck, shoulder, and lumbar back pain in the participants (yes = 0, no = 1) as the dependent variable, and the factors with significant differences in the single-factor analysis as the independent variable. The results suggested that the frequency of occurrence of chronic neck, shoulder, and lumbar back pain in medical personnel was correlated with the extent of anxiety, frequency of bending over in the past 1–2 years, whether taking relevant measures to prevent neck, shoulder, and lumbar back pain, gender, whether they had a senior professional designation, the time spent working with the head tilted down at a desk each day, and whether they underwent training to prevent neck, shoulder, and lumbar back pain in their department. The details are presented in Table 4.

**Table 4:** The results of the multiple-factor logistic regression analysis

Variate		Regression coefficient	Standard error	Z value	P value	OR (95%CI)
Intercept		-3.272	0.422	-7.747	< 0.001	0.038 (0.017–0.087)
Extent of anxiety	High VS Low	2.683	0.321	8.370	< 0.001	14.634 (7.807–27.432)
Frequency of bending over in the past 1–2 years	High VS Low	2.049	0.250	8.202	< 0.001	7.762 (4.756–12.666)
Taking relevant measures to prevent neck, shoulder, and lumbar back pain at work	Yes VS No	0.711	0.242	2.935	0.003	2.036 (1.266–3.274)
Gender	Female VS Male	0.823	0.284	2.904	0.004	2.278 (1.307–3.971)
Professional title	Junior					1 (Reference)
	Intermediate	0.182	0.245	0.744	0.457	1.200 (0.743–1.938)
	Deputy senior	0.469	0.305	1.540	0.123	1.599 (0.880–2.905)
	Senior	1.104	0.527	2.094	0.036	3.016 (1.073–8.472)
	Others	-2.381	1.293	-1.842	0.066	0.092 (0.007–1.165)
The amount of time spent working with the head down at a desk each day	High VS Low	0.495	0.209	2.370	0.018	1.641 (1.089–2.471)
Having training about how to prevent neck, shoulder, and lumbar back pain in their own department	Yes VS No	-0.651	0.323	-2.017	0.044	0.521 (0.277–0.982)

## Discussion

### The morbidity of chronic neck, shoulder, lumbar, and back pain among medical personnel was high

The results showed that 588 participants had neck, shoulder, and lumbar back pain to different degrees in the past 1–2 years, and the occurrence rate was 97.7%. Which indicated that medical workers are a high-risk group for chronic neck, shoulder, and lumbar back pain. Our findings were similar to those of other studies [11–13], which was probably because the participants were from a large tertiary general hospital within the province. These hospitals have many patients, heavy workloads, and long working hours. Also, the job responsibilities of the hospital are complex, and medical technology-related and logistical positions involve varying degrees of damage to the lumbar back muscles, eventually leading to chronic lumbar back pain. Long-term chronic neck, shoulder, and lumbar back pain might cause anxiety and depression, which can affect life and quality of work. Even worse, it might cause cervical spondylosis and lumbar spondylosis in medical personnel, which can severely affect their physical and psychological health. Thus, we recommend that hospitals should comprehensively consider the working conditions and individual problems of the staff and take effective measures to decrease the rate of incidence of chronic neck, shoulder, and lumbar back pain.

### Chronic neck, shoulder, and lumbar back pain positively correlated with the ODI score

The ODI is the most frequently used assessment tool to evaluate the dysfunction of patients with lumbar back pain in rehabilitation medicine, spine surgery department, and other fields; the higher the ODI score, the more serious the dysfunction [10]. Our results indicated a positive correlation (*r* = 0.574, *P* < 0.001) between the ODI score and the frequency of neck, shoulder, and lumbar back pain (points 1–5), similar with prior research [14–16]. These findings suggested that the higher the frequency of chronic lumbar back pain occurring within the same period, the longer the duration of pain, and the higher the ODI score. Thus, the lumbar back pain occurring in medical personnel also requires attention. If individuals experience pain frequently within a short period, they should be given timely treatment.

### Analysis of the risk of chronic neck, shoulder, and lumbar back pain among medical personnel

In this study, we selected medical personnel from a tertiary general hospital in Zunyi City as participants, and identified the factors associated with chronic neck, shoulder, and lumbar back pain experienced by medical personnel after the outbreak of the COVID-19 pandemic. Our findings indicated that the extent of anxiety, frequency of bending over in the past 1–2 years, whether individuals took relevant measures to prevent neck, shoulder, and lumbar back pain at work, gender, with senior professional title, the time spent working with the head tilted down at a desk each day, and whether they underwent training to prevent neck, shoulder, and lumbar back pain in their departments were factors associated with the suffering of medical personnel due to chronic neck, shoulder, and lumbar back pain (*P* < 0.05).

### Anxiety

We found an independent and interactional relationship between anxiety and chronic pain. Long-term chronic pain can cause different degrees of mental and psychological disorders, such as nervousness, anxiety, fear, and depression. Additionally, mental and psychological disorders can aggravate pain, resulting in persistent pain that can recur frequently [17, 18]. Our findings suggested that the risk of having chronic neck, shoulder, and lumbar back pain in people with a high degree of anxiety was 14.634-fold higher than that for people with a low degree of anxiety. Hu’s research shows that patients with chronic low back pain were more likely to experience anxiety symptoms in China [19]. Darnall and Ziadni also propose that anxiety has a role in pain initiation [20, 21]. Since the outbreak of the COVID-19 pandemic, these medical workers have been overburdened with clinical work under high-pressure conditions; they also bear social and family pressure, which makes them prone to mental health problems [22]. Hence, the mental health conditions of medical personnel need to be checked regularly, and psychological guidance or training should be provided if required, to ease occupational stress and decrease the incidence of pain to some extent.

### Bending over and related intervening measures

Our results indicated that the risk of having chronic neck, shoulder, and lumbar back pain in participants who needed to bend over frequently was 7.76-fold (95% CI: 4.756–12.666) higher than that for the participants who bent over rarely. Medical technology workers often need to lower their heads and bend over to perform various examinations, move patients, and perform various medical nursing operations for patients, such as lowering their heads and bending over, standing up for surgery, moving equipment, etc. Some medical workers often participate in repairing and also assist in handling heavy physical tasks. Regardless of their behavior, such activities have a high chance of causing lumbar spine injuries [12]. Long-term bending leads to local stress aggregation in the body, which increases pressure and causes strain in the lumbar muscles. Frequent bending also increases the pressure on the lumbar intervertebral disc, which can easily lead to chronic tearing of the annulus fibrosus, causing degenerative changes in the nucleus pulposus tissue and symptoms such as the tension of the lumbar muscles and radiating pain in the legs. The risk of having chronic neck, shoulder, and lumbar back pain in people who took prevention measures at work was 2.036 fold (95% CI:1.266–3.274) higher than those who did not, indicating that, some medical personnel took measures to prevent chronic neck, shoulder, and lumbar back pain but eventually achieved a counterproductive outcome. This occurred probably because the measures they took were either insufficient or the methods were inappropriate, and also because they had excessive work intensity. The risk of having chronic neck, shoulder, and lumbar back pain in participants who received training on the prevention of neck, shoulder, and lumbar back pain in their respective departments was 0.521-fold (95% CI: 0.277–0.982) lower than that for the patients who did not receive training. Thus, hospitals need to increase training and spread awareness on the prevention of chronic neck, shoulder, and lumbar back pain among non-specialist personnel, train the staff on the basic theory and practice of human mechanics, guide medical personnel to lift heavy objects based on human mechanics, correct bad posture, and teach the personnel correct prevention measures, to effectively reduce the incidence of chronic neck, shoulder, and lumbar back pain [23].

### Gender

Among the 602 participants in this study, 478 participants, (79%) were female, and 124 participants, (21%) were male, the number of female participants was significantly greater than the number of male participants. The risk of suffering from neck, shoulder, and lumbar back pain was 2.278 times (95% CI: 1.307–3.971) higher among females than males, which was probably related to the bias in the ratio of females to males in this study; However, some studies [24, 25] have shown that the reproductive history is associated with the occurrence of lumbar back pain. The physical characteristics related to reproduction in females changes that increase the stress on their lumbar and back during pregnancy. Most women of reproductive age also undertake household and childcare responsibilities along with their professional work, which further increases their risk of developing lumbar back pain compared to the risk in males.

***Professional title and the amount of time spent working at a desk each day with the head tilted down***.

We found that the higher professional designation, the longer time working with the head tilted down at a desk, and the higher the risk of suffering from neck, shoulder, and lumbar back pain. Our findings matched those of a study by Fan [26]. Such a pattern occurred probably because of several reasons. First, the participants in this study were from a tertiary general hospital in Zunyi City, where medical personnel experience high pressure at work, and must undertake heavy teaching and research activities along with daily medical work. Additionally, the requirements for medical personnel for receiving promotion are very strict. Thus, medical staff need to work very hard for promotion. Also, the higher the designation, the greater the responsibility and pressure. Second, medical personnel have to spend their free time after work learning new things, which further increases their time at the desk every day, and the longer the time they spend working at a desk each day, the longer the time they maintain the same posture, the heavier the stress on the spine, and the higher the risk of suffering from neck, shoulder, and lumbar back pain.

## Conclusion

Medical personnel are a high risk of developing chronic neck, shoulder, and lumbar back pain. Hospitals and departments need to increase awareness of related knowledge, while individuals need to make reasonable use of their time and exercise their neck, shoulder, lumbar, and back muscles regularly. They should also keep warm, avoid cold, and participate in various sports activities, such as Tai Chi, aerobics, yoga, etc., to improve their physical fitness. There are several limitations to our research. First, the cross-sectional design and small sample size are limitations that must be noted when interpreting the findings because they impair the study’s generalizability. Second, as the sample was from one institution, further study on more hospital staff is required to achieve more reliable results.

## Data Availability

All data generated and analyzed during this study are included in this article.

## Abbreviations

ODI: The Oswestry disability index
AIC: Akaike’s information criterion
CI: Confidence Interval
